# CalR and MPL Driver Mutations and Their Role in the Diagnosis and Clinical Course of JAK2-Unmutated Chronic Myeloproliferative Neoplasm: Results from a Pilot Single-Center Study

**DOI:** 10.3390/medicina61060962

**Published:** 2025-05-23

**Authors:** Tarık Onur Tiryaki, Aynur Dağlar Aday, Meliha Nalçacı, Akif Selim Yavuz

**Affiliations:** 1 Faculty of Medicine, Department of Internal Medicine, Division of Hematology, Istanbul University, Istanbul 34134, Turkey; mnalcaci@istanbul.edu.tr (M.N.); yavuzas@istanbul.edu.tr (A.S.Y.); 2 Faculty of Medicine, Department of Internal Medicine, Division of Medical Genetics, Istanbul University, Istanbul 34134, Turkey; aynur.aday@istanbul.edu.tr

**Keywords:** myeloproliferative disorders, primary myelofibrosis, essential thrombocythemia

## Abstract

*Background and Objectives*: Philadelphia (Ph)-negative myeloproliferative neoplasms can exhibit defects in Janus kinase 2 (JAK2), Calreticulin (CalR), and MPL genes. It is possible that the presence of other driver mutations may influence diagnosis and prognosis in patients who do not have a JAK2 gene mutation. The purpose of this study was to assess the frequency of CalR and MPL gene mutations and the clinical effects of these mutations in JAK2 gene-unmutated MPN patients from a single center. *Materials and Methods*: We examined 46 patients (ET/PMF: 34/12) diagnosed with MPNs regarding their genetic conditions, diagnoses, and complications. *Results*: CalR Type 1 gene mutation was detected in 26.1% of cases, CalR Type 2 gene mutation in 13.0%, MPL-L gene mutation in 2.2%, and MPL-K gene mutation in 6.5%. In total, 56.5% of patients were triple-negative. The presence of CalR Type 1 and Type 2 mutations was significantly more prevalent in patients with essential thrombocytosis (ET), although the difference did not reach statistical significance (*p* = 0.51, *p* = 0.57). In contrast, MPL mutations were only observed in patients with primary myelofibrosis (PMF). *Conclusions*: We found no correlation between thrombosis, leukemic transformation, and driver mutations. MPL gene mutation was present in only myelofibrosis patients, and CALR gene mutation was present in one of the three cases of leukemic transformation. The triple-negative group had a lower survival rate, but this difference was not statistically significant (110.3 months vs. 121.4 months, respectively, *p* = 0.53). However, the sample size was quite small. Our limited observations suggest a possible trend that requires confirmation.

## 1. Introduction

Philadelphia (Ph)-negative myeloproliferative neoplasms (MPNs) are defined as diseases resulting from abnormal proliferation of terminal myeloid cells in peripheral blood. MPNs are a group of diseases resulting from clonal expansion of cells that can differentiate into mature cells in one or more myeloid lineages. In 1951, Dameshek grouped CML, PV, ET, and PMF under MPNs [1]. Classical Ph-negative MPN subtypes include polycythemia vera (PV), essential thrombocythemia (ET), and primary myelofibrosis (PMF). A combination of gene polymorphisms, JAK2, Calreticulin, and MPL driver mutations, and clinical parameters can predict the prognosis and clinical course (overt myelofibrosis or conversion to acute leukemia, thrombosis, and significant bleeding) [2,3,4].

It may be challenging to determine the subtype of MPN in patients without the JAK2 gene mutation. CalR and MPL gene mutations may be helpful in making the correct diagnosis [5]. During the course of the disease, complications such as thrombotic/thromboembolic events, severe bleeding, post-PV or -ET myelofibrosis, or acute leukemia may develop [6,7,8]. The course of the disease can be predicted based on these mutations. For example, patients with CalR gene mutations have higher platelet counts and fewer thrombotic complications. Fibrosis is more commonly associated with MPL gene mutations. It is known that the survival rate is lower in the groups that do not possess these mutations, known as triple-negative (TN). Consequently, it is important to investigate other driver mutations in addition to the JAK2 gene mutation. Detection of MPN-associated mutations is crucial for patients, but routine laboratory testing is costly and workflows can delay diagnosis. This study investigated the frequency of CalR and MPL gene mutations in Bcr-Abl-negative chronic myeloproliferative patients without the JAK2 mutation and their possible effects, using a simple and effective technique, high-resolution melting (HRM).

## 2. Materials and Methods

### 2.1. Patients

This study was approved by the Institutional Ethics Committee of Istanbul University’s Faculty of Medicine Ethics Committee on 15 August 2018. The ethical code for this study is 2018/461. It followed the procedures outlined in the Helsinki Declaration 1975, as revised in 2000. Patients provided written informed consent before samples were obtained. Data and patients were retrospectively selected from the Department of Hematology at the Istanbul Faculty of Medicine. The primary objective of this study was to evaluate the demographic characteristics, survival, disease course, and status of CalR and MPL gene mutations in patients with JAK2-unmutated Ph-negative MPNs at our center. Between March 2004 and January 2013, due to a limited number of test kits, the first 46 consecutive patients were included. Patients whose diagnoses were confirmed according to WHO criteria, who did not carry the JAK2 mutation, and who had regular follow-up were chosen for the patient selection. In total, 46 patients with ET and PMF were enrolled (28 females and 18 males with a mean age of 53.5 years, range 23–93 years). Participants in this study were over 18 years of age with a diagnosis of ET or PMF by WHO criteria. This pilot study provided preliminary observations that need to be confirmed in larger cohorts.

### 2.2. JAK2, CalR, and MPL Gene Analysis

For each patient, genomic DNA was isolated from peripheral blood collected in a tube containing EDTA. The concentration of the isolated DNA samples was determined with a Nanodrop 2000c spectrophotometer (Thermo Scientific, Wilmington, DE, USA). JAK2 V617F mutation was analyzed using a commercial kit (JAK2 MutaScreen assay; Ipsogen, Marseille, France). It was analyzed with a cut-off value of 2%. Patients with a VAF value < 2% were considered JAK2 gene-unmutated. CalR and MPL gene mutation status was evaluated in PMF or ET patients without the JAK2 mutation.

CalR (exon 9) and MPL W515 L/K (exon 10) mutations were analyzed on a LightCycler 480II Real-Time PCR device (Roche, Rotkreuz, Switzerland). Probe-based PCR and Sanger sequencing are relatively expensive, while next-generation sequencing (NGS) is both expensive and labor-intensive, requiring a laboratory workflow with a long turnaround time. High-resolution melting (HRM) is a simple and effective technique. So, CalR mutation analysis was performed using high-resolution melting (HRM) analysis, and MPL W515L/K mutation analysis was performed using HRM and allele-specific PCR analysis. Regarding the reliability of this study, each sample was studied twice. The samples were divided into groups by comparing the differences in the melting curves obtained from the analysis. Samples that differed in melting curves in the analysis were considered potential mutation carriers. If a CalR mutation was detected, TaqMan-based analysis was used for verification. It has been reported to have a detection limit of up to 3% for CalR and MPL mutations [9]. In light of this situation, 3% was chosen as the threshold value.

### 2.3. Statistical Analysis

We performed all statistical analyses using SPSS software (version 25.0, SPSS, Chicago, IL, USA). Descriptive statistics (mean, standard deviation, median, frequency, ratio, minimum, and maximum) were used to evaluate the data. Kolmogorov–Smirnov and Shapiro–Wilk tests were used to confirm the normality of the quantitative data. The Mann–Whitney U test compares two groups of non-normally distributed data. Non-normally distributed parameters were compared intra-group using the Wilcoxon Signed Rank test. Fisher’s Exact test was used to compare qualitative data. In this study, overall survival (OS) is defined as the period between the time of diagnosis and death for whatever reason. The Kaplan–Meier method was used to evaluate OS. There was a significance level of at least *p* < 0.05.

## 3. Results

Forty-six patients who were diagnosed with JAK2 V617F-unmutated chronic myeloproliferative Bcr-Abl-negative disease between March 2004 and January 2013 were included in this study; 73.9% (*n*: 34) of all patients had ET and 26.1% (*n*: 12) had PMF. All PV patients were carrying the JAK2 gene mutation, so they were omitted. The ages of the cases ranged between 23 and 93 (28 females and 18 males, with a mean age of 53.5 years).

Table 1 summarizes the baseline characteristics of all patients.

CalR Type 1 gene mutation was detected in 26.1% (*n* = 12) of cases, CalR Type 2 gene mutation in 13.0% (*n* = 6), MPL-L gene mutation in 2.2% (*n* = 1), and MPL-K gene mutation in 6.5% (*n* = 3), as shown in in Table 2. The rate of CalR gene mutation in patients with PMF who do not have the JAK2 gene mutation was 41.6%. On the other hand, the rate of MPL gene mutation in this population was 33.3%. In the ET group that had unmutated JAK2, the CalR mutation rate was 35.2%, and the MPL mutation was not detected. CalR Type 1 and MPL-L gene mutations were detected in one patient with PMF. CalR Type 1 and CalR Type 2 gene mutations were detected in one patient with ET. Twenty-six (%56.5) patients were negative for CalR Type 1, Type 2, and MPL L/K gene mutations. There was no statistically significant difference between the rates of incidence of CalR Type 1, CalR Type 2, and MPL-L gene mutations in the two disease groups (*p* > 0.05), but there was a statistically significant difference between the incidence rates of MPL-K gene mutation in these groups (*p* = 0.003; *p* < 0.05). At the same time, it is noteworthy that none of the patients with ET had MPL-L or MPL-K mutations.

The patients were evaluated regarding their mutational status and age; there was no statistical significance between age and CalR Type 1 and 2 gene mutations in patients with essential thrombocythemia (*p* = 0.62, *p* = 0.81, respectively). Similarly, no significant difference was found between the gene mutations and age in patients with PMF (CalR Type 1 gene mutation *p* = 0.71, MPL-K gene mutation *p* = 0.30). The mutational status for CalR Type 2 and MPL-L were not statistically significant. CalR Type 1, MPL-L, and age statistically significantly increased the risk of disease. Gender and CalR Type 2 and MPL-K are not significant, as shown in Table 3.

Patients with PMF had a mean follow-up period of 99 months (1–175 months), whereas those with ET had a mean follow-up period of 120 months (59–243 months) (*p* = 0.27). No effect of mutation status on survival was observed in our subgroup analyses. In this study, we also did not observe significant differences in overall survival between patients in the triple-negative group and others (110.3 months vs. 121.4 months, respectively, *p* = 0.53). In this period, leukemic transformation was observed in 3 of 46 patients (*n*: 2 (%5.9) ET, *n*: 1 (%8.3) PMF patients). CalR gene mutation was present in one ET patient with leukemic transformation, while the other two patients were triple-negative. In Table 4, the results are summarized. In six patients, major thrombotic events were observed. Three patients had CalR mutations, two had CalR Type 1, and one had CalR Type 2, as shown in Table 5. There was a higher mortality rate in patients with PMF, regardless of mutational status (%58.3 vs. %14.7, *p* = 0.006).

## 4. Discussion

In chronic myeloproliferative disorders with Bcr-Abl negativity, the JAK2 gene mutation is the most common mutation (accounting for 95% of PV cases, 60% of ET cases, and 50% of PMF cases). In 2016, the WHO emphasized the importance of the CalR and MPL gene mutations in hematopoietic and lymphoid tissue tumor classification and in the diagnosis of PMF and ET [2,4]. There have been recommendations since then to look for three main driver mutations during diagnosis.

Many studies and the revised WHO criteria have shown that the JAK2 V617F, CalR, and MPL gene mutations are crucial for the diagnosis of chronic myeloproliferative neoplasms. The frequency of driver mutations increases with increasing age and disease progression. Driver mutations and germline polymorphisms can predict whether patients have essential thrombocythemia, polycythemia vera, or myelofibrosis. Approximately 20% of ET patients and 10 to 15% of PMF patients have no mutation in these three driver genes. These patients are called triple-negative patients (TNs) [2].

Obtaining genetic test results in a short time frame is critical for the management of MPN patients. Compared to TaqMan probe-based RT-PCR, digital PCR (dPCR), Sanger sequencing, and NGS methods, HRM is highly advantageous due to its low cost, relatively low complexity, high reliability, and particularly fast turnaround time, requiring much less expensive instrumentation and reagents. Furthermore, the sensitivity of HRM is up to 97%. HRM analysis uses the difference in melting temperatures between unique sequence models to detect a variant sequence in the background of the wild-type sequence, and does not require any post-PCR processing [9].

In this study, we investigated the frequency of CalR and MPL gene mutations using the HRM method at the time of diagnosis. We evaluated their contributions to clinical effects during follow-up. Statistical significance was not found due to the small sample size, but the results may be clinically valuable. Genomic data can be integrated with clinical parameters to predict patient outcomes [10,11].

In ET patients, the JAK2 V617F driver mutation is found in approximately 60%, while CalR and MPL mutations are found in approximately 20% and 3%, respectively [12,13]. CalR gene mutation is found in 20–80% of ET patients without JAK2 and MPL gene mutations. In our study, the rate of CalR gene mutation in ET without the JAK2 mutation was found to be similar to the rates in the literature (35.2%).

It is well known that patients diagnosed with ET carrying an MPL mutation have the worst prognosis [14]. However, among our ET patients, none of the patients were found to carry an MPL mutation. Due to this, we were unable to reach a conclusion regarding our own patients.

According to our current knowledge, the CalR gene mutation is associated with a younger age and a higher platelet count [10]. In ET patients, the CALR gene mutation appears to be associated with a reduced risk of thrombosis. There are many factors that contribute to the development of thrombosis, including congenital (hereditary thrombophilia) and acquired factors (such as antiphospholipid syndrome, inflammation, atherosclerosis, the JAK2 mutation, and MPN-associated thrombosis), that may be frequently observed. The JAK2 gene mutation is associated with an increased risk of thrombosis [14,15,16]. Fatal and nonfatal thrombotic events occur in 1.9% of patients per year [17]. Half of our six patients who developed thrombosis during follow-up (all in the ET group) had mutations in the CalR gene (two with CalR Type 1 mutation and one with CalR Type 2).

There are various reports of progression to accelerated phase at 10 years, ranging from 0.7% to 1.9% [13,18]. ET with CalR gene mutations progressed to blastic or accelerated phases more frequently than ET with JAK2 mutations [5]. Leukemic transformation was observed in one patient with the CalR gene mutation in our groups. Triple-negative patients are at greater risk of leukemic transformation and have worse outcomes [13,19].

Type 2 CalR gene mutations cause more fibrosis than Type 1 CalR gene mutations. CalR Type 2 mutations are also associated with higher platelet counts than CalR Type 1 mutations in ET patients [10,20]. CalR Type 2 mutations tend to be associated with ET, whereas Type 1 mutations are associated with PMF [2]. In our group, CalR Type 1 gene mutations were more common in PMF cases.

PMF harbors 52–67% JAK2 V617F mutation, 4–12% CalR gene mutation, and 4–6% MPL mutation [21]. CalR mutations in the PMF group were associated with lower leukocyte count, lower bone marrow cellularity, and more megakaryocytes. On the other hand, a study in patients with PMF showed that the median survival time in patients with the CalR gene mutation was significantly longer than in those without the CalR mutation. This correlation was not detected in our study; this may be related to the size of the population. It is noteworthy that no thrombosis or leukemic transformation occurred in our PMF group.

A reduction in total and erythroid bone marrow cellularity was observed in MPL-mutated patients, whereas there was a significant increase in the number of megakaryocytes, megakaryocytic clusters, and small megakaryocytes. MPL gene mutation has been reported in %5–10 patients with JAK2-negative ET or PMF [14]. The incidence of the MPL gene mutation was statistically higher in our patients with PMF. The difference between ET and profibrotic MF might be challenging in some cases. The presence of atypical megakaryocytes, granulocytic proliferation, and clinical symptoms such as increased LDH or splenomegaly supports a diagnosis of prefibrotic PMF. It is distinguished from ET by abnormally large and dense megakaryocyte clusters. MPL gene mutation may be more valuable in patients with a suspicious diagnosis, especially post-ET myelofibrosis patients. In our patients, it was noteworthy that no MPL mutation was observed in any patient with ET. It is possible that, in rare instances, the presence of the MPL mutation could be a beneficial indicator of PMF.

MPN is a genetically heterogeneous disease consisting of germinal and somatic mutations that contribute to the disease. This heterogeneity can be demonstrated using next-generation sequencing techniques. Studies have shown that atypical JAK2, CALR, and MPL variants are essential for diagnosing these diseases [22]. It has been shown that there is a high rate of poor prognostic mutations, such as SH2B3 and ASXL1, in the presence of driver mutations containing these atypical variants [22,23]. Due to this situation, sequencing techniques for new generations have become more critical.

There are some limitations to this article. The small number of patients and the inability to obtain a sufficient statistically significant interpretation are important factors. The most important aspect of the model is that it is able to predict the diagnostic process and the clinical course in the current rare disease group, that it is compatible with the literature, and that it reflects long-term follow-up data. Despite this, there is no further insight to be gained from the numerical data because of the small sample size. Additionally, in the long term, complications such as transformation to leukemia, thrombosis, and fibrosis should also be accounted for by other acquired factors (inflammation, individual characteristics, and comorbidities).

## 5. Conclusions

This article shows that some mutations are more common in some disease groups. We found that MPL gene mutations were only observed in PMF patients in our study; however, further research is needed to improve diagnosis, treatment, and prognosis in MPNs. There was a higher incidence of leukemia transformation and thrombosis in patients carrying mutant CalR genes in our group. Nevertheless, these data have not been statistically proven and need to be verified. Gene mutations at the time of diagnosis are important for predicting the changes that will be important in such long-term follow-ups. Therefore, identification of these three most common driver mutations is very important for making a correct diagnosis or predicting disease progression. It may be helpful during the diagnostic process to look for mutations other than the JAK2 gene mutation, so that the most appropriate method may be used to guide the diagnosis and course of treatment. This pilot study provided preliminary data. Further research is expected to contribute to the diagnosis, treatment, and prognosis of MPN.

## Figures and Tables

**Table 1:** Main demographic and hematological features of 46 patients with JAK2-unmutated Bcr-Abl-negative chronic myeloproliferative disease.

Variable	Total MPN (*n*: 46)	PMF (*n*: 12)	ET (*n*: 34)	*p*
**Male/female, % male**	18/28 (39.1)	2/10 (16.6)	16/18 (47.0)	*0.06*
**Age (onset), y**	53.5 (23–93)	55.1 (33–85)	53 (23–93)	*0.97*
**Leucocytes (×10^9^/L)**	8.9 (3.6–24.5)	9.4 (3.6–23.4)	8.8 (4.9–24.5)	*0.80*
**Hemoglobin (g/dL)**	11.6 (5.8–15.9)	9.7 (5.8–11.1)	12.3 (7.9–15.9)	***0.001***
**Platelets (×10^9^/L)**	857 (147–2631)	533 (147–921)	993 (326–2631)	***0.001***

**Table 2:** CalR and MPL gene mutation status in patients with PMF and ET.

Gene Mutation	PMF (*n* = 12, %)	ET (*n* = 34, %)	MPN (*n* = 46, %)	*p*
**CalR-Type 1**	4 (33.3)	8 (23.5)	12 (26.1)	*0.51*
**CalR-Type 2**	1 (8.3)	5 (14.7)	6 (13.0)	*0.57*
**MPL-L**	1 (8.3)	0 (0)	1 (2.2)	***0.09***
**MPL-K**	3 (25.0)	0 (0)	3 (6.5)	***0.*** ***003***

**Table 3:** Logistic regression analysis of impact of CalR and MPL mutations on disease risk.

Variable	Unadjusted OR	Adjusted OR	*p*-Value
**CalR Type 1**	1.50	1.57 (1.04–2.36)	**0.04**
**CalR Type 2**	0.80	0.74 (0.49–1.12)	0.25
**MPL-L**	1.01	1.02 (1.01–1.03)	**0.02**
**MPL-K**	0.98	0.99 (0.97–1.01)	0.34
Age	1.04	1.05 (1.00–1.10)	**0.02**
**Gender (male)**	1.70	1.82 (0.93–3.58)	0.10

**Table 4:** Patients with leukemic transformation: characteristics and treatment.

Leukemic Transformation					
	Age at the Diagnosis and Sex	Diagnosis, Follow-Up (Months)	Age and Diagnosis at the Transformation	Mutation Status	Treatment and Status
Case 1	62, M	ET, 150	75, AML	CalR Type 1 mutated	Hydroxyurea, Exitus in 4th month
Case 2	68, M	ET, 83	76, CMML-AML	Triple-Negative	Azacitidine, Exitus in the second month
Case 3	53, F	PMF, 160	70, AML	Triple-Negative	Azacitidine, Exitus in the third month

AML: acute myeloid leukemia; CMML: chronic myelomonocytic leukemia.

**Table 5:** Patients with major thrombotic events: characteristics and treatment.

Major Thrombotic Events							
	Age at the Diagnosis and Sex	Diagnosis, Follow-Up (Months)	Age at Thrombosis	Mutation Status	Thrombosis	Pre-Thrombotic Treatment	Treatment and Status
Case 1	39	ET, 134	39	Triple-Negative	Portal vein thrombosis	None	Hydroxyurea and warfarin, Alive
Case 2	31	ET, 84	33	CalR Type 1 mutated	Portal vein thrombosis	Acetylsalicylic acid	Hydroxyurea and warfarin, Alive
Case 3	52	ET, 77	52	Triple-Negative	Ischemic stroke	None	Hydroxyurea and acetylsalicylic acid, Alive
Case 4	93	ET, 88	95	CalR Type 1 mutated	Myocardial infarction	Interferon-alpha and acetylsalicylic acid	Interferon-alpha and acetylsalicylic acid, Exitus
Case 5	60	ET, 205	64	Triple-Negative	Portal vein thrombosis	Hydroxyurea and acetylsalicylic acid	Hydroxyurea and warfarin, Alive
Case 6	65	ET, 129	68	CalR Type 2 mutated	Portal vein thrombosis	Hydroxyurea and acetylsalicylic acid	Hydroxyurea and warfarin, Alive

## Data Availability

The datasets used and/or analyzed in the current study are available from the corresponding author upon reasonable request.
